# Information Retrieval Using Machine Learning for Biomarker Curation in the Exposome-Explorer

**DOI:** 10.3389/frma.2021.689264

**Published:** 2021-08-19

**Authors:** Andre Lamurias, Sofia Jesus, Vanessa Neveu, Reza M. Salek, Francisco M. Couto

**Affiliations:** ^1^ LASIGE, Departamento de Informática, Faculdade de Ciências, Universidade de Lisboa, Lisbon, Portugal; ^2^ International Agency for Research on Cancer, Lyon, France

**Keywords:** machine learning, text mining, information retrieval, biomarkers of exposure, database curation

## Abstract

**Objective:** In 2016, the International Agency for Research on Cancer, part of the World Health Organization, released the Exposome-Explorer, the first database dedicated to biomarkers of exposure for environmental risk factors for diseases. The database contents resulted from a manual literature search that yielded over 8,500 citations, but only a small fraction of these publications were used in the final database. Manually curating a database is time-consuming and requires domain expertise to gather relevant data scattered throughout millions of articles. This work proposes a supervised machine learning pipeline to assist the manual literature retrieval process.

**Methods:** The manually retrieved corpus of scientific publications used in the Exposome-Explorer was used as training and testing sets for the machine learning models (classifiers). Several parameters and algorithms were evaluated to predict an article’s relevance based on different datasets made of titles, abstracts and metadata.

**Results:** The top performance classifier was built with the Logistic Regression algorithm using the title and abstract set, achieving an F2-score of 70.1%. Furthermore, we extracted 1,143 entities from these articles with a classifier trained for biomarker entity recognition. Of these, we manually validated 45 new candidate entries to the database.

**Conclusion:** Our methodology reduced the number of articles to be manually screened by the database curators by nearly 90%, while only misclassifying 22.1% of the relevant articles. We expect that this methodology can also be applied to similar biomarkers datasets or be adapted to assist the manual curation process of similar chemical or disease databases.

## 1 Introduction

Biomarkers are biological parameters objectively measured in the body as indicators of normal biological conditions, environmental lifestyles, pathological conditions, or responses to therapeutic interventions (Strimbu and Tavel, 2010). They can be chemicals, metabolites, enzymes or other biochemical substances, like products of an interaction between a compound and a target molecule or a cell type. Characterizing the relationship between biomarkers and the possible biological outcomes is crucial to correctly predict clinical responses, screen, monitor and diagnose patients and to improve efficiency in clinical trials. Biomarkers play a significant role in risk assessment, as they allow one to identify exposure to hazards and to associate responses with the probability of a disease or exposure outcome.

Biomarkers of exposure are a specific type of biomarkers that reflect exposure of an individual to an environmental factor (such as diet, pollutants or infectious agents) known to affect the etiology of diseases. Compounds can get in contact with living organisms through absorption, inhalation or ingestion and then are either metabolized, stored or eliminated. This exposure can be detected by analysing biospecimens, such as blood or urine, or by measuring concentrations and characterizing the exogenous substance, its metabolites or its products of interaction with target molecules.

Exposomics is the study of the totality of exposures of a particular individual over lifetime, from an omics perspective. In the recent years, several studies have focused on studying the exposome, since this a new paradigm to biomedical informatics (Martin Sanchez et al., 2014). For example, Kiossoglou et al. (2017) present an approach to analyse exposome studies based on word frequency counts and ontology concepts. They applied their methodology to a set of 261 abstracts, and identified terms, concepts and ontologies to characterize the current knowledge about the human exposome. Lopez-Campos et al. (2019) expanded this methodology to more documents, and found that exposomics research and literature doubled over the course of 2 years (2016–2018), and identified more relevant ontologies.

The Exposome-Explorer is the first database dedicated to biomarkers of exposure for environmental risk factors for diseases released in 2016 (Neveu et al., 2016) and updated in 2020 (Neveu et al., 2020) based at the International Agency for Research on Cancer, part of the World Health Organization. Exposome-Explorer is a highly curated resource by experienced researchers working in the exposomics field. The database contents resulted from a manual literature search that yielded over 8,500 citations, but only a small fraction of these publications were used in the final database. Manually curating a database is time-consuming and requires domain expertise to gather relevant data scattered throughout millions of articles. This work proposes a supervised machine learning pipeline, trained based on the existing curation work to update the resource with new information using a literature retrieval mechanism and manual curation.

Gathering relevant data scattered throughout millions of articles from text repositories is a time-consuming task which requires specialized professionals to manually retrieve and annotate relevant information within the articles. Keeping biological databases updated as new papers are released, as well as collecting new data, is equally challenging and time consuming. Such tasks would benefit from being assisted with text-mining tools. To our knowledge, there is no Information Retrieval (IR) solution available to assist literature screening regarding biomarkers of exposure using machine learning.

Studies have been carried out to either improve the IR task using machine learning or to perform entity recognition (ER) and information extraction (IE) on biomarker data. However, none applies IR-based methods to biomarkers of exposure. Almeida et al. (2014) developed a machine learning system for supporting the first task of the biological literature manual curation process, called triage, which involves identifying very few relevant documents among a much larger set of documents. They were looking for articles related to characterized lignocellulose-active proteins of fungal origin to curate the mycoCLAP database (Strasser et al., 2015). They compared the performance of various classification models, by experimenting with dataset sampling factors and a set of features, as well as three different machine learning algorithms (Naïve Bayes, Support Vector Machine and Logistic Model Trees). The most fitting model to perform text classification on abstracts from PubMed was obtained using domain relevant features, an under-sampling technique, and the Logistic Model Trees algorithm, with a corresponding F-measure of 0.575. Lever et al. (2019) used a supervised learning approach to develop an IE-based method to extract sentences containing relevant relationships involving biomarkers from PubMed abstracts and Pubmed Central Open Access full text papers. With this approach, they built the CIViCmine knowledge base, containing over 90,992 biomarkers associated with genes, drugs and cancer types. Their goal was to reduce the time needed to manually curate databases, such as the Clinical Interpretation of Variants in Cancer (CIViC) knowledgebase (Griffith et al., 2017), and to make it easier for the community curators, as well as editors, to contribute with content.

Following these previous approaches, this work aims to reduce the time, effort and resources necessary to keep the Exposome-Explorer database updated as new articles are published, by using a supervised machine learning approach to automatically classify relevant publications and automatically recognize candidate biomarkers to be reviewed by the curators. The approach of the curators of this database consisted in developing search queries to retrieve relevant publications and then manually analyse each one. However, the number of publications retrieved is still too large to manually screen each one of them. This work proposes a system, available on Github (https://github.com/lasigeBioTM/BLiR), to further narrow down the literature that holds important information about biomarkers of exposure. The existing manually curated data used to develop the Exposome-Explorer database has been used to train and test the models (classifiers). We also provide a corpus of articles classified by our system as relevant, along with biomarkers automatically annotated on the abstracts of these articles. When given a new publication, these classifiers can predict whether this publication is relevant to the database and annotate the candidate biomakers mentioned on that document.

## 2 Methods

### 2.1 Exposome-Explorer Dataset

This work was developed using the data used to set up and develop the Exposome-Explorer, which included:

− the queries used to search for citations with information about dietary and pollutant biomarkers in the Web of Science (WOS), which are provided as additional data;
− the WOS search results based on the previous queries, with 8,575 citations used to manually screen the relevant articles containing biomarker information;
− the 480 publications used to extract information about biomarkers for the database.

**FIGURE 1:**
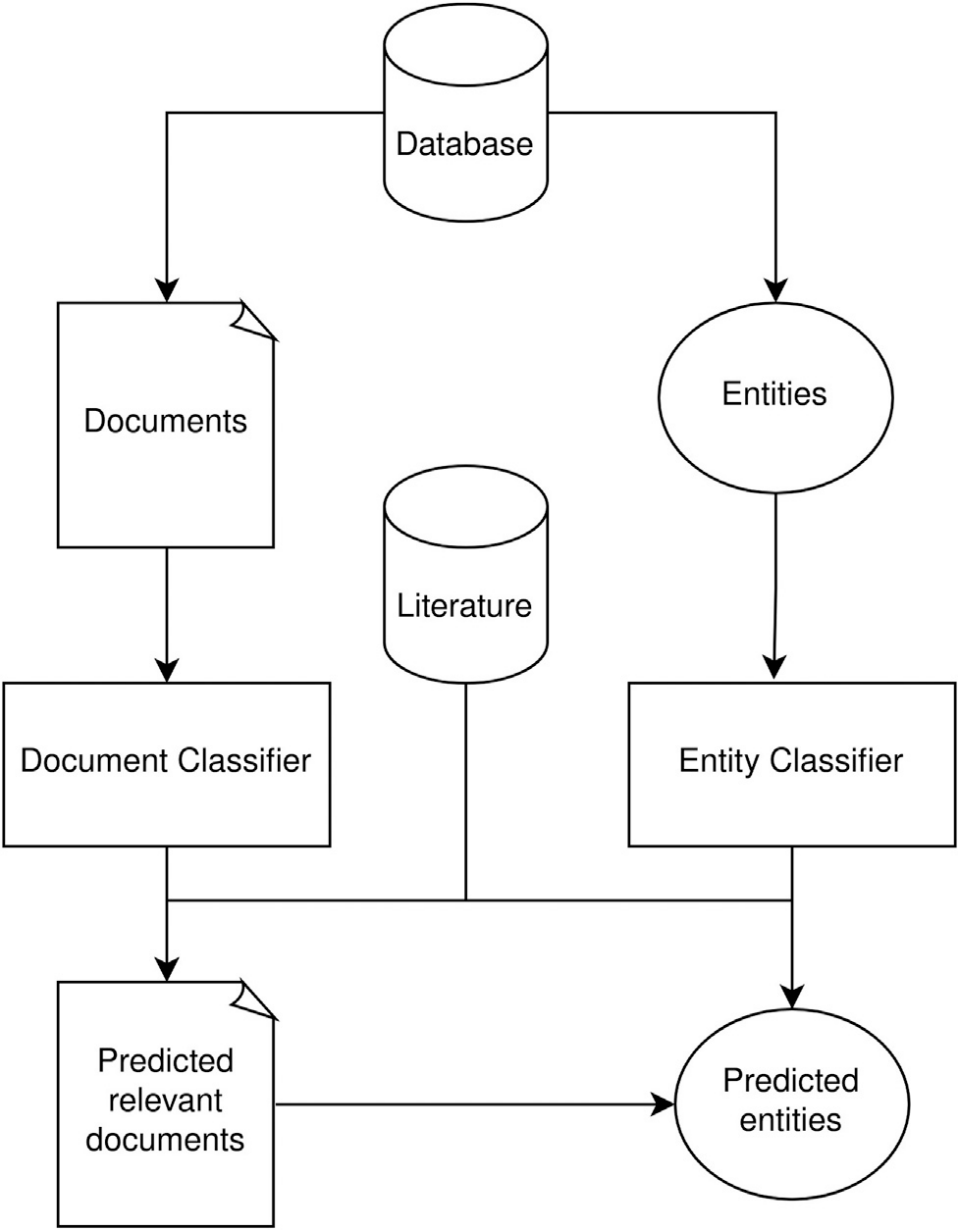
Diagram of the Information Retrieval workflow for database curation. Using existing curated information from a database, it is possible to train both document and entity classifiers. These classifiers can then generate new candidate entries based existing literature.

Figure 1 shows a general workflow of our pipeline. In this case, we can start with a first version of the database, such as is the case of Exposome-Explorer. The documents used to develop the database can then be used to train classifiers that are able to predict other relevant documents, while the entry names can be used to train entity recognition classifiers to predict new candidate entries.

### 2.2 Data Collection

All 480 publications used to curate the database were expected to be listed in the 8,575 citations retrieved from the WOS. However, only 396 of them were present: the 84 publications absent from the WOS query results were additionally identified by database annotators while screening the literature for relevant articles. These 84 scientific papers were excluded from the dataset used to build the models since we could not replicate the original workflow if we included them.

The main objective of this database was to gather information about concentration values of biomarkers in various human biospecimens and correlation values between dietary intakes and biomarker concentrations. Therefore, the papers used to curate the database reflect this criteria. In the version of the database used for this dataset, the search results of the queries related to dietary biomarkers were explored more exhaustively, therefore providing more consistent labels. The curators added publications that mentioned correlations between specific biomarker measurements and food or dietary compound intake, therefore establishing a more defined type of relevant article. For this reason we consider both a scenario where only papers relevant to dietary biomarkers, and where all types of papers were considered.

The existing dataset, listed above, was missing some features that we wanted to explore to construct our models, such as number of citations and PubMed ID. For this reason, PubMed was used to extract the titles, abstracts and metadata (publication date, author names, number of times the article was cited and journal name). The PubMed search and retrieval of PMIDs, titles, abstract and metadata was carried out with E-utilities, a public API available at NCBI Entrez system. Some publications were found through the DOI to PMID (PubMed ID) converter and others by a combined search with the title and first author name. The resulting corpus of articles consisted of 7,083 publications.

### 2.3 Data Preprocessing

After retrieving the title, abstract and metadata for each article, it was necessary to prepare the textual data to be used as input by the machine learning models (classifiers). This task included:

1. *Assign labels to each article:* A supervised learning approach was used to build the classifiers, which means each article (document) has a known class assigned to it. To label each article, the list with the 396 articles used to curate the database was cross-referenced with the 7,083 publications in the corpus. If they were present in the list of 396 database articles, they were considered relevant and assigned the label 1. If they were not present in the list, as they were not used to extract information about biomarkers, they were considered irrelevant and therefore assigned the label 0;
2. *Text pre-processing and tokenization:* The text was separated into words (tokens). All words with the same root were reduced to a common form (stemming) and all stop words were removed. The tokens were lowercased and then combined into n-grams, a contiguous sequence of *n* items from a given sample of text or speech. For example, for *n* = 2, the features from the sentence “*Determining thiocyanate serum levels*,” were combined into three n-grams: “*determin thiocyan*,” “*thiocyan serum*” and “*serum level*;”
3. *Transform textual data to numerical data:* The machine learning model expects numeric data as its input. However, the titles, abstracts, and metadata are in text format. To this end, each distinct token that occurred in the documents was mapped to a numerical identifier, and the number was used to refer to the token instead of the token itself;
4. *Build the matrices:* Each feature represents one column and each document represents a row of the matrix. Depending on the type of matrix chosen, the matrix contained either n-gram counts (number of times each term occurs in each document) or TFIDF (term frequency-inverse document frequency) features (how important a n-gram is to a document in a collection of documents). An additional column was added to the training and testing data, with the respective labels. The goal of the classifier was to predict this column when applied to a new data.

The metadata of each article was handled slightly differently from the titles and the abstracts. Since it already had numerical attributes (publication date and number of citations), the matrix was created with two columns dedicated to these features, instead of having one column for each year and number of citations. The authors’ names were joined into one single word (Wallace RB → WallaceRB) and were neither combined into n-grams nor went through the stemming and stop word removal stages. The journal name had no special preprocessing.

Stemming was performed using the class SnowballStemmer from the module nltk. stem in the NLTK package (Loper and Bird, 2002). Steps (2), (3) and (4) used the Scikit-learn (Pedregosa et al., 2011) classes CountVectorizer and TfidfVectorizer from the module sklearn. feature_extraction.text. The main difference between the two classes is that the first one converts a collection of raw text documents to a matrix of token counts and the last one to a matrix of TFIDF features. Combinations of three different parameters were tested to preprocess the data, resulting in different matrices used to build the classifier and, therefore, different results. The parameters tested were:

−ngram_range (min_n, max_n): the lower and upper boundary of n for different n-grams to be extracted. The range values tested were *n* = {1}, *n* = {1, 2} and *n* = {1, 3};
− min_df: ignore all n-grams that have a document frequency lower than the given threshold. If min_df = 2, then terms that only appear in one article (document) will be ignored. The values of min_df ranged from 2 to 23, depending on the value of n used in the ngram_range parameter ([1 + *n* − *gram*, 21 + *n* − *gram*]);
− type of the matrix: matrix of token counts or TFIDF features.

Finally, we divided the dataset into a train set of 70% and a test set of 30%, while keeping the same proportion of positive and negative classes on both subsets. The train set was used to optimize the parameters through 10-fold Cross Validation (CV) and the test set was used to obtain the results on held-out data.

### 2.4 Machine Learning Models

The goal of the IR task was to reduce the time needed to screen the articles, by narrowing down the literature available to a set of publications that provide a reliable resource of information, in this specific case, related to biomarkers of exposure. Thus, in this case we can model the IR task as a classification task, where we have to decide whether a document is relevant or not.

#### 2.4.1 Building the Classifiers

The machine learning models, also known as classifiers, were separately trained and tested using the titles, abstracts, titles + abstracts and titles + metadata, to assess which section of the article was more suitable to predict its relevance. We explored the combination of titles and metadata since our preliminary results indicated that the metadata by itself would not obtain reasonable results. However, these preliminary results also indicated that combining the abstracts with metadata would result in equal or worse results than using just the abstracts. For this reason, we did not explore the option of combining abstracts with metadata, or combining all three.

Six machine learning algorithms were explored:

• Decision Tree (Apte et al., 1998): the features are fractioned in branches that represent a condition to be applied to each instance;
• Logistic Regression (Walker and Duncan, 1967): learns a logistic function to perform binary classification;
• Naïve Bayes (Zhang, 2004): the independence of the features is assumed and a probability model is used to determine the most probable label for each instance;
• Neural Network (Rumelhart et al., 1986): this algorithm can learn non-linear functions by introducing hidden layers between the input features and output label;
• Random Forest (Breiman, 2001): combines various tree estimators trained on subsamples of the training data;
• Support Vector Machine (Cortes and Vapnik, 1995): the data is represented as points in a hyperplane and the algorithm tries to establish a clear division between the instances with the same label.

The Scikit-learn package was used to run these algorithms. Most of the parameters used for each algorithm were the default ones, however, a few ones were altered to better suit the data (class_weight, solver, kernel, gamma, bootstrap, n_estimators), others to maximize the performance of the model (C, alpha, max_depth, min_samples_leaf), and one to assure a deterministic behaviour during fitting (random_state). The values of the parameters altered to maximize the performance of the model were found through grid search with 10-fold CV on the train set. Table 1 summarizes the Scikit-learn functions used and the parameters changed for each algorithm.

**TABLE 1:** Scikit-learn functions and parameters for each algorithm: Decision Tree (DT), Logistic Regression (LR), Naïve Bayes (NB), Neural Network (NN), Random Forest (RF) and Support Vector Machine (SVM).

	Sklearn functions	Parameters
DT	DecisionTreeClassifier	class_weight = “balanced;” random_state = 0; min_samples_leaf = 5
LR	LogisticRegression	class_weight = “balanced;” random_state = 0; solver = “liblinear;” C = 10.0, 1.0 or 0.1^a^
NB	MultinomialNB	alpha = 0.01
NN	MLPClassifier	solver = “lbfgs;” random_state = 0
RF	RandomForestClassifier	class_weight = “balanced;” random_state = 0; bootstrap = False; max_depth = 20; min_samples_leaf = 2; n_estimators = 100
SVM	SVC	class_weight = “balanced;” random_state = 0; kernel = “rbf;” gamma = “scale”

a *C* = 0.1 for term-count matrices; For TFIDF matrices, *C* = 10.0 for the abstracts; *C* = 1.0 for the titles and titles + metadata; *C* = 0.1 for the metadata.

#### 2.4.2 Ensemble Learning

When testing different classifiers using the abstracts, titles, titles + abstracts or the titles + metadata set, the prediction each model makes for a certain article might differ. The titles + metadata model may correctly identify a publication as being relevant, while the abstracts model fails to do so. For this reason, we explored ensembles of classifiers to understand if we could retrieve more relevant publications this way.

We used two ensemble approaches to join the results of multiple models. The first was Bagging, where the same algorithm is used to train a classifier on random subsets of the training data, and the results are then combined (Breiman, 1996). The second was Stacking, which consists in training multiple classifiers and using their output to train a final model which predicts the classes (Wolpert, 1992). With this approach, each of the first-level classifiers can be specified, as well as the final classifier. Therefore, we used all of the previously mentioned algorithms as first-level classifiers, and then tried each of them as the final estimator. For the Bagging approach, we also tried every algorithm previously mentioned. In both cases, we used the parameters specified in Table 1, using the Scikit-learn implementations and the default parameters of the BaggingClassifier and StackingClassifier classes.

### 2.5 Performance Evaluation

In the data preprocessing task, labels were given to each article: 0 for irrelevant (negative) and one for relevant (positive). These labels were considered the gold-standard and represent the actual class of the publications.

In the document classification task, all classifiers built were optimized using the Scikit-learn CV function (sklearn.model_selection.cross_validate). This model optimization technique provides a more accurate estimate of the model’s performance, since it evaluates how the model will perform on unseen data. Additionally, we selected a test set to evaluate the models after parameter optimization.

The cv parameter of the function determines how many groups the data will be split into. In this work, a *cv* = 10 was used, which means the data was separated into 10 groups, each one was used 9 times as a training set and once as the testing set. Ten different models were built using the same parameters, with different training sets. Each time a trained model was applied to testing data, it generated a vector with predicted classes for those documents. By comparing the predictions of the testing set to the gold standard, it was possible to separate the documents into four different categories:

− True Positives (TP): documents correctly labelled as positive;
− False Positives (FP): documents incorrectly labelled as positive;
− True Negatives (TN): documents correctly labelled as negative;
− False Negatives (FN): documents incorrectly labelled as negative.

This categorization allows to calculate the precision and recall, two commonly used metrics that assess the performance of the tools by measuring the quality of the results in terms of relevancy. Precision (P) is the proportion of true positives items over all the items the system has labelled as positive. Recall (R) is the proportion of true positives items over all the items that should have been labelled as positive.

$$P=\frac{TP}{TP+FP}\;\;\;\;\;\;\;\;\;\;\;\;\;\;\;\;R=\frac{TP}{TP+FN}$$

The F1-score is a measure between 0 and one that combines both precision and recall. Higher values of this metric indicate that the system classifies most items in the correct category, therefore having low numbers of FP and FN.

$$F1=2\cdot \frac{P\cdot R}{P+R}$$

Furthermore, we also considered a variation of the F1-score, the F2-score, where more weight is given to the recall:

$$F2=5\cdot \frac{P\cdot R}{4\cdot P+R}$$

This metric was important for our evaluation since we wanted to avoid low recall values, which would mean that many documents were mistakenly classified as not relevant. Our objective was to reduce the number of documents that manual curators had to analyse, but without losing important information, therefore preferring false positives over false negatives. This evaluation strategy has also been used in other document curation studies (Gay et al., 2005; Almeida et al., 2014; Rae et al., 2019).

To estimate the balance between the true positive rate (recall) and false positive rate, we also computed the AUC (Area under the ROC Curve), using the Scikit-learn implementation of this measure that computes the area under a curve plotted by the true positive rate and false positive rate at various thresholds.

### 2.6 Biomarker Recognition

We performed biomarker recognition on the documents classified as positive by our best performing classifier. The objective of this task was to demonstrate how the document classifiers can be used to aid the curation process. By automatically screening the articles for biomarkers, curators can focus on articles that mention entities of their interest and help them to extract information from those articles.

To train a NER classifier, it is necessary to have a dataset where the words corresponding to the entities of interest are annotated. Since we did not have this type of dataset for biomarkers, we developed our own training set based on the biomarkers of the Exposome-Explorer database. We identified all the biomarker names of the database on the documents using MER, a Minimal Entity Recognition tool (Couto and Lamurias, 2018). This tool returns a list of entities recognized in the text, including their exact location and unique identifier, if available. The resulting dataset will not be as gold standard, however these automatically generated datasets have been shown to be enough to train information extraction models in some cases (Rebholz-Schuhmann et al., 2010; Sousa et al., 2019). Furthermore, we trained our model using a Transformer architecture (Vaswani et al., 2017), based on a pre-trained model for the biomedical domain (Gu et al., 2021). This way we only had to fine-tune the pre-trained model on the biomarker entities from our dataset.

We evaluated the NER classifier similarly to the document classifiers, using F1-score, precision and recall, although we only evaluated on a held-out test set. We trained for 10 epochs using the default parameters of the Transformers library^1^. Afterwards, we run the trained model on the documents that were not used to create the database, in order to find potential candidate entries that might have been missed.

## 3 Results

### 3.1 Data Collection and Preprocessing

After data collection, the Exposome-Explorer dataset consisted of titles, abstracts, and metadata from a total of 7,083 publications. Among them, 6,687 were considered irrelevant, because no information about biomarkers was extracted from them for the Exposome-Explorer database. The remaining 396 publications were considered relevant, as they were used to construct the database.

In the beginning, all articles from all types of biomarkers in the dataset were used, however, this approach yielded poor results. To try to improve the results, the data was restricted to articles obtained using queries specific to dietary biomarkers, since they were handled more attentively by the curators. The new dataset consisted of 3,016 publications (2,860 irrelevant +156 relevant).

### 3.2 Document Classification

#### 3.2.1 Dietary Biomarker Publications

Our first objective was to train models to classify which articles from a search query were relevant to the Exposome-Explorer database. We optimized both the parameters used to preprocess the diet training data (ngram, minimum frequency, vectorizer), as well as hyperparameters of each algorithm, using grid search-CV. For each algorithm, we tested several combinations and selected the trained models that achieved the highest score of each metric on the CV evaluation.

The maximum values each algorithm could reach for these metrics, using optimized preprocessing and algorithm parameters, are summarized in Table 2. The complete values for each highest metric, as well as the parameters used, can be found in Additional File 1. For example, the maximum F2-score of 0.701 of the LR algorithm on the titles + abstracts set was obtained using a min_df of 5, ngram_range (1, 3) and a token count matrix. We can see that all algorithms except Decision Trees could achieve high values on the various data subsets, although using only the titles, the LR algorithm achieved higher scores in most metrics. The parameters and algorithms used to maximize the F2-score for each feature set can be found in Table 4.

**TABLE 2:** Dietary biomarkers document classification results. Highest precision, recall, F1-score, F2-score and AUC achieved by each algorithm: Decision Tree (DT), Logistic Regression (LR), Naïve Bayes (NB), Neural Network (NN), Random Forest (RF) and Support Vector Machine (SVM). The highest value of each metric on each feature type is bolded.

TITLES					
	MaxPrecision	MaxRecall	MaxF1	MaxF2	Max-AUC
DT	0.216	0.433	0.262	0.302	0.635
LR	0.388	**0.707**	**0.495**	**0.601**	**0.910**
NB	0.528	0.652	0.475	0.561	0.903
NN	0.560	0.331	0.385	0.348	0.887
RF	0.415	0.661	0.489	0.577	0.889
SVM	**0.586**	0.688	0.462	0.560	0.904

ABSTRACTS					
	MaxPrecision	MaxRecall	MaxF1	MaxF2	Max-AUC
DT	0.337	0.570	0.397	0.449	0.720
LR	0.559	0.770	0.618	**0.687**	0.953
NB	0.606	0.752	**0.644**	0.684	0.952
NN	**0.854**	0.441	0.542	0.472	0.948
RF	0.621	0.705	0.591	0.644	**0.954**
SVM	0.512	**0.798**	0.572	0.658	0.949

TITLES + ABSTRACTS					
	MaxPrecision	MaxRecall	MaxF1	MaxF2	Max-AUC
DT	0.379	0.533	0.419	0.480	0.740
LR	0.550	0.779	0.614	**0.701**	0.948
NB	0.601	0.788	**0.643**	0.695	0.950
NN	**0.764**	0.432	0.528	0.463	0.945
RF	0.665	0.687	0.586	0.634	**0.953**
SVM	0.512	**0.807**	0.564	0.664	0.948

TITLES + METADATA					
	MaxPrecision	MaxRecall	MaxF1	MaxF2	Max-AUC
DT	0.307	0.468	0.328	0.390	0.693
LR	0.392	**0.734**	**0.502**	**0.616**	**0.930**
NB	0.373	0.707	0.456	0.566	0.914
NN	**0.603**	0.266	0.333	0.274	0.905
RF	0.476	0.725	0.492	0.592	0.924
SVM	0.163	0.168	0.144	0.154	0.725

The highest value of each metric is bolded.

In addition to exploring single classifiers, we also explored two ensemble approaches: Bagging and Stacking. We trained a Stacked classifier that combined the best individual models (Table 1), and then applied again one of the algorithms as the final classifier. Table 3 show the maximum Precision, Recall, F1-Score, F2-score and AUC of each algorithm, using the Stacking and Bagging approach, and training only on the abstracts + titles subset, which provided the best results of most of the individual models. This way, we can compare directly with the results of Table 2. The full set of values of each metric is also provided in Additional File 1.

**TABLE 3:** Dietary biomarkers ensemble classifiers’ results. Highest precision, recall, F2-score and AUC reached for each algorithm: Decision Tree (DT), Logistic Regression (LR), Naïve Bayes (NB), Neural Network (NN), Random Forest (RF) and Support Vector Machine (SVM). The NB algorithm did not work with the Stacking approach.

BAGGING					
	MaxPrecision	MaxRecall	MaxF1	MaxF2	Max-AUC
DT	**0.742**	0.477	0.546	0.502	0.941
LR	0.664	**0.707**	**0.642**	**0.663**	0.950
NB	0.716	0.580	0.594	0.582	0.951
NN	0.729	0.477	0.537	0.497	0.942
RF	0.648	0.541	0.571	0.550	**0.958**
SVM	0.740	0.405	0.489	0.433	0.957

STACKING					
	MaxPrecision	MaxRecall	MaxF1	MaxF2	Max-AUC
DT	0.417	0.735	0.513	0.622	0.842
LR	0.380	**0.890**	0.521	**0.685**	**0.961**
NN	**0.581**	0.505	0.521	0.509	0.911
RF	0.568	0.734	**0.624**	0.673	0.952
SVM	0.374	**0.890**	0.513	0.679	0.947

The highest value of each metric is bolded.

**TABLE 4:** Algorithm and parameters used to get the highest F2 for each set of data.

	Title	Abstracts	T + a	T + M
Algorithm	LR	LR	LR	LR
df	4	4	5	4
n-gram	[1, 2]	[1, 3]	[1, 2]	2
matrix	token-count	TFIDF	token-count	token-count
Precision	0.388	0.500	0.514	0.392
Recall	0.707	0.770	0.779	0.734
F1-score	0.495	0.587	0.614	0.502
F2-score	0.601	0.687	0.701	0.616
ROC AUC	0.910	0.938	0.937	0.930

We then applied the classifiers of the previous table with highest F2-score to the test set which we did not use during grid search-CV 5. With this held-out dataset, we wanted to observe if the classifiers had been overfitted to the training set due to the parameter optimization procedure.

#### 3.2.2 All Biomarker Publications

To quantify how much restricting the dataset to dietary biomarkers had improved the results, new models were trained with the whole corpus of 7,083 publications from all biomarkers using the same algorithms and parameters that had maximized the recall score for dietary biomarkers. The comparison between the values of precision, recall and F-score can be found in Table 6.

### 3.3 Biomarker Entity Recognition

We trained a NER model for biomarkers using a silver standard corpus which we provide along with the code. On a held-out set of 30% of the sentences, we obtained an F1-score of 0.6735, for a precision of 0.5574 and recall of 0.8507. However, this evaluation was made on automatically annotated data, and as such there is a high chance that some of the false positives that lowered the precision were due to entities that were not annotated in the silver standard.

We then applied this model on 7,444 documents that were not used to develop the database and that we had title and abstract available. We aggregated the extracted entities, filtered out entities that were already entries in Exposome-Explorer and manually validated the top 100 entities that appeared more often. We observed that 45 of these entities were biomarker names that were not already present in the database. These entities constitute new candidate entries, along with the supporting documents from where they were found.

## 4 Discussion

The highest F2-score (0.701) was obtained using a single classifier with the Logistic Regression (LR) algorithm on the abstracts and title set, using cross-validation (Table 2). Among the 905 dietary publications used to test the classifiers, 365 were classified as positive, which could reduce by 90% (proportion of articles classified as positive) the time needed to find 77.9% (recall score) of the relevant articles, and only 22.1% of the relevant articles would be lost. Looking at the results from the titles and metadata set, globally lower values were obtained when compared to the abstracts sets. Using features from both the titles and abstracts resulted in better F2-scores in almost every algorithm, comparing with using them separately. This indicates that, similarly to how it is carried out during manual curation, both titles and abstracts should be considered when evaluating the relevance of an article to the database. The LR algorithm obtained the best performance on many metrics, although the SVM algorithm obtained a higher recall using the titles and titles and abstracts, and Random Forests obtained the highest AUC on the same sets. The Neural Networks algorithm obtained the highest precision using the abstracts, titles and abstracts, and titles and metadata sets.

**FIGURE 2:**
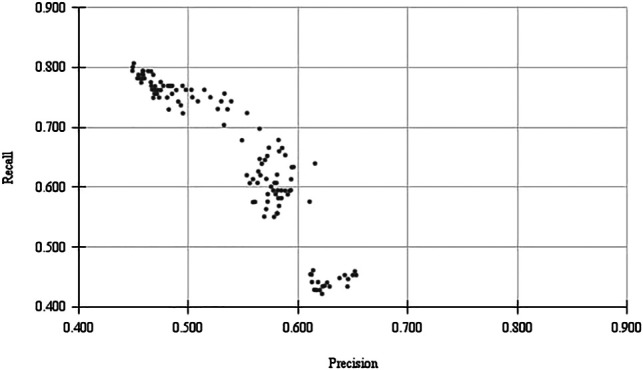
Trade-off between Precision and Recall for different parameters of a classifiers trained with Naive Bayes.

The balance between precision and recall is an important topic to take into consideration in this type of approach. We included both the balanced F1 scores and the F2 scores, as both have been used in other document curation studies. However, we cannot say for sure the balance that is expected by a curator. While ideally no relevant document should be excluded by a classifier (False Negatives), it will make the curators task harder if many irrelevant documents are presented (False Positives). Figure 2 illustrates how just one algorithm can obtain a range of P/R trade-offs. With the same algorithm (Naïve Bayes), we can train a classifier more biased for Precision or Recall. A user-study could help understand what is the balance that is more ideal for database curators.

To assess whether joining the best models would improve the scores, we applied two ensemble approaches to the abstracts set: Bagging and Stacked. In some cases, using a Bagging approach results in better scores than just the model by itself, for example, comparing the scores of the Decision Tree classifier. However, in most cases, using just one classifier provided better results. The Stacking approach also obtained better scores in some cases, including a maximum recall of 0.890 using the Logistic Regression and SVM classifiers. However this approach took much longer to train since it requires training one model with each of the previously mentioned algorithms, as well as an additional model to predict the class based on the other models’ prediction scores. Furthermore, both ensemble approaches resulted in similar or worse results than the single classifiers. This could be due to the increased complexity of these models, which may be less adaptable to new data due to overfitting to the train data.

In Table 5, we can see the effect of the cross-validation evaluation when compared to the test set validation. Although some of the scores are lower, the LR algorithm achieves the highest balanced scores and the Neural Networks achieves the highest precision. The Stacking algorithm achieves a high recall, but at the cost of lower precision. Although the balanced metrics are lower on the test set when compared to the test set evaluation, we believe that the difference is not relevant, since the cross-validation results were averaged over five iterations, and the test set shows the results of only one run.

**TABLE 5:** Dietary biomarkers classifiers on the test set. Precision, recall, F2-score and AUC achieved each algorithm: Decision Tree (DT), Logistic Regression (LR), Naïve Bayes (NB), Neural Network (NN), Random Forest (RF) and Support Vector Machine (SVM), as well as the Bagging and Stacking approaches, using the combinations that achieved the highest F2-score.

	Precision	Recall	F1	F2	AUC
DT	0.403	0.532	0.459	0.500	0.744
LR	0.530	0.745	**0.619**	**0.689**	0.854
NB	0.515	0.745	0.609	0.684	0.853
NN	**0.700**	0.447	0.545	0.482	0.718
RF	0.450	0.766	0.567	0.672	0.857
SVM	0.451	0.787	0.574	0.685	0.867
Bagging	0.542	0.681	0.604	0.648	0.825
Stacking	0.388	**0.851**	0.533	0.687	**0.889**

The highest value of each metric is bolded.

### 4.1 Error Analysis

In order to interpret the gap of results between the training set and the predictions obtained from the classifiers on each cross-validation iteration, the LR classifier built with the titles was analysed. This classifier had a similar recall score to the abstracts but, as the titles are shorter, they make the interpretation easier. One interesting pattern we noticed was that almost all titles that had the words “food frequency questionnaire” were classified as relevant. As previously mentioned in section 2.2, this related to how articles about dietary biomarkers were chosen to be included in the database. From a total of 82 titles containing these words, only 2 were classified as irrelevant (both had words such as “calcium”, “water” and “energy” that were mostly found on irrelevant articles); 29 were TP and the remaining 51 were being wrongly labelled as relevant.

The title “Toenail selenium as an indicator of selenium intake among middle-aged men in an area with low soil selenium” was classified as negative, when it was in fact used in the database (FN). 39 out of 40 titles with the word “selenium” were not used in the database and thus labelled irrelevant: this over-represented feature may be the reason why the classifier failed to classify this article as relevant although selenium was considered of interest by the annotators.

It is also important to highlight that papers inserted in the database have been analysed considering the full-texts. This means that papers tagged as “relevant” either by the classifier and/or manually, could subsequently be rejected by the annotators, for a variety of reasons including “the paper is not-available online,” or “the data in the paper is not presented in a way acceptable for the database.” These papers would then be considered false positive by the classifier, because they are present in the corpus of citations but absent from the database.

Restricting the analysis to the dietary biomarker citations provided much better metrics than when using all the data from the database (dietary, pollutants, and reproducibility values) (Table 6). When restricting the analysis to citations describing the different classes of biomarkers of pollution, the performance of the models was even lower (preliminary results not shown). This difference in performance could be explained by the difference of nature of the data searched by the annotators for the different sets of biomarkers. For dietary biomarkers, the focus was made on publications providing correlation values between dietary intakes and biomarkers measured in human biospecimens, and mostly describing validation studies of dietary questionnaires with biomarkers. For the pollutant biomarkers, the focus was made on papers describing concentration values of pollutant biomarkers in human biospecimens. Moreover, by lack of time and human resources, not all potentially relevant publications on pollution biomarkers were inserted in the Exposome-Explorer, while the dietary biomarkers were handled more attentively. As a consequence, the dietary biomarkers account for almost half of the entries of the database. All of this could explain why the model seems to perform better for dietary biomarkers. Having a closer look at false positives obtained by the classifier on pollutants could be a good way to check if the model developed on dietary biomarkers could also be applied to pollutants, and identify new relevant papers from the corpus of pollutants. This also means that as we obtain a more comprehensive corpus for other classes of biomarkers, the performance of our machine learning solution will also improve.

**TABLE 6:** Comparison of precision, recall and F-score between the whole dataset and the restricted dataset.

TITLES					
	Precision	Recall	F1	F2	ROC AUC
All biomarkers	0.362	0.529	0.430	0.485	0.737
Dietary biomarkers	0.386	0.574	0.462	0.523	0.762

ABSTRACTS					
	Precision	Recall	F1	F2	ROC AUC
All biomarkers	0.218	0.765	0.340	0.510	0.801
Dietary biomarkers	0.342	0.809	0.481	0.635	0.862

TITLES + ABSTRACTS					
	Precision	Recall	F1	F2	ROC AUC
All biomarkers	0.354	0.630	0.453	0.545	0.781
Dietary biomarkers	0.468	0.787	0.587	0.693	0.869

TITLES + METADATA					
	Precision	Recall	F1	F2	ROC AUC
All biomarkers	0.310	0.681	0.426	0.550	0.795
Dietary biomarkers	0.330	0.638	0.435	0.538	0.784

### 4.2 Biomarker Recognition

To demonstrate how biomarker recognition can also be useful for database curation, we trained a model based on the entities and documents that we already in the database, in order to find biomarkers and documents that might have been missed during the curation process. We extracted a total of 7,444 entities, however many of these entities were incomplete or duplicated. We then looked at the top 100 entities that appeared most often, excluding entity names that were already in the database, and found 45 potential new entries, which we provide as Supplementary Data Sheet. Of these, we highlight sucrose, which is described as a biomarker along with fructose (Tasevska et al., 2005). Although fructose exists in Expose-Explorer, sucrose was missing. A NER model like the one we trained could have prevented this. Another example is Julin et al. (2011), an article that studies the role of cadmium as a biomarker, which was also missed during the development of the database.

## 5 Conclusion

The Exposome-Explorer database is being manually curated, without any assistance from machine learning tools. As the number of scientific papers continues to grow, text-mining tools could be a great help to assist the triage of documents containing information about biomarkers of exposure and keep the database updated.

To this end, several machine learning models were created using different combinations of preprocessing parameters and algorithms. These classifiers were trained using the publications’ abstracts, titles and metadata. The model with the highest F2-score (70.1%) was built with the LR algorithm and used the titles and abstracts to predict a paper’s relevance. We also extracted named-entities from the abstracts, obtaining a total of 45 candidate biomarkers.

To apply this methodology to the database curation pipeline, the IR task will consist of two steps. In the first one, articles will be retrieved using the query search on WOS, to target domain-specific publications. Then, the classifier could be used to narrow down the publications even more, and a named-entity recognition tool can be used to provide candidate entries to the database. Manual curation will still be needed, to extract information about biomarkers from full-text articles.

In the future, we will work on improving the results from the classifiers that use the metadata set. For example, by assigning different weights to the authors, according to the position they appear in, or by creating new features that result from the combinations between all authors within the same article. We will also study the impact of the recognized biomarkers in the retrieval classification. When analysing why the model misclassified some publications, a few chemicals, like “calcium” and “selenium” were strongly associated with irrelevant articles. An idea to explore is to replace chemical tokens by a category they belong, such as “chemical,” and see if it improves the precision and recall of the classifiers. This should avoid over-fitting on the training set. Furthermore, we will improve this gold standard by adding more types of biomarkers, that can also classify non-dietary biomarkers. Another idea to explore is to train deep learning models for document classification, even though this would require more training data and will take longer to train than the algorithms used in this paper. Finally, the performance of classifiers trained on this dataset when applied to the results of other search queries will be explored.

## Data Availability

The datasets presented in this study can be found in online repositories. The names of the repository/repositories and accession number(s) can be found below: https://github.com/lasigeBioTM/BLiR.

## Footnotes

1
https://github.com/huggingface/transformers
